# Evolutionary selection of a 19-stranded mitochondrial β-barrel scaffold bears structural and functional significance

**DOI:** 10.1074/jbc.RA120.014366

**Published:** 2020-08-19

**Authors:** Shashank Ranjan Srivastava, Radhakrishnan Mahalakshmi

**Affiliations:** Molecular Biophysics Laboratory, Department of Biological Sciences, Indian Institute of Science Education and Research, Bhopal, India

**Keywords:** porin, VDAC, voltage gating, 19-stranded barrel, structure–function relation, mitochondria, outer membrane protein, ion channel, voltage-dependent anion channel (VDAC), membrane protein, outer membrane, spectroscopy, circular dichroism (CD), beta-barrel

## Abstract

Transmembrane β-barrels of eukaryotic outer mitochondrial membranes (OMMs) are major channels of communication between the cytosol and mitochondria and are indispensable for cellular homeostasis. A structurally intriguing exception to all known transmembrane β-barrels is the unique odd-stranded, *i.e.* 19-stranded, structures found solely in the OMM. The molecular origins of this 19-stranded structure and its associated functional significance are unclear. In humans, the most abundant OMM transporter is the voltage-dependent anion channel. Here, using the human voltage-dependent anion channel as our template scaffold, we designed and engineered odd- and even-stranded structures of smaller (V2^16^, V2^17^, V2^18^) and larger (V2^20^, V2^21^) barrel diameters. Determination of the structure, dynamics, and energetics of these engineered structures in bilayer membranes reveals that the 19-stranded barrel surprisingly holds modest to low stability in a lipid-dependent manner. However, we demonstrate that this structurally metastable protein possesses superior voltage-gated channel regulation, efficient mitochondrial targeting, and *in vivo* cell survival, with lipid-modulated stability, all of which supersede the occurrence of a metastable 19-stranded scaffold. We propose that the unique structural adaptation of these transmembrane transporters exclusively in mitochondria bears strong evolutionary basis and is functionally significant for homeostasis.

Cellular homeostasis requires mitochondrially generated ATP to be transported seamlessly from the inner mitochondrial compartments to the cytosol. For decades, it was believed that the outer mitochondrial membrane (OMM) was leaky and passively conducted ions and metabolites between the cytosol and mitochondrial inner membrane. The discovery of porins in the OMM in 1976 (1) established, for the first time, the existence of membrane proteins that actively regulate the bidirectional flux of ATP as well as other metabolites, proteins, lipids, cholesterol, and ions, thereby allowing mitochondria to efficiently establish cellular homeostasis (2–5). Porins, known in higher eukaryotes as voltage-dependent anion channels (VDACs), adopt transmembrane β-barrel structures. They have now been recognized as active cell regulators, indispensable elements for mitochondrial bioenergetics, vital checkpoints for communication of mitochondria with the cytosol and endoplasmic reticulum, and in mitochondria-mediated apoptosis (3–8).

From the structural standpoint, three other transmembrane β-barrels in addition to VDACs have been characterized thus far in the OMM, namely, Tom40, Sam50, and Mdm10 (in lower eukaryotes) (9). These mitochondrial β-barrel outer membrane proteins (mOMPs) constitute ∼1% of the total human proteome), are found exclusively in the OMM, and were believed to have endosymbiotic origins (9). The first structures of isoform 1 of human and mouse VDACs resolved to atomic resolution in 2008 (10–12) revealed the most unexpected finding that unlike the even-stranded porins pervasive in bacteria (13), VDACs adopt a unique β-barrel structure with an odd number of β-strands, *i.e.* 19 β-strands. The complete structural characterization of isoform 2 of zebrafish VDAC (14) and *Neurospora* Tom40 (15) and the modeled structure of Mdm10 (16) further confirmed that all three mOMP β-barrels are 19-stranded, with a α-helix at the N terminus. Additionally, these three β-barrels have no established bacterial ancestor (17, 18).

The structural and evolutionary significance of the unique scaffold of mOMPs has been an enigma since the first structural characterization, because odd-stranded barrels are conspicuously absent in their bacterial ancestors. Barrel closure in these structures is achieved through parallel H-bonding between the 1st and 19th strands, which could render this scaffold structurally weaker than antiparallel H-bonding (19). Moreover, the functional requirement of an odd-stranded barrel has remained a recurring controversy (20, 21). It is known that the structure and stability of integral membrane proteins such as VDACs are intricately connected with its lipidic membrane. Although mitochondria have evolved by endosymbiosis of archaic bacteria, the OMM architecture is remarkably distinct from its ancestor. More specifically, the lipid composition of the mitochondrial outer membrane of mammalian cells is enriched with phosphatidylcholines (PCs) that constitute >50% w/w of the total OMM lipid, whereas PC lipids are inexistent in the outer membrane of Gram-negative bacteria (22, 23). Hence, we reasoned that the biogenesis and evolutionary selection of a 19-stranded β-barrel over an 18- or 20-stranded counterpart coevolved for functional superiority in the unique PC-enriched mitochondrial lipid milieu and should link its unique structure to a functional role specific to the microenvironment of the mitochondrial outer membrane.

In OMPs with an odd number of strands, the first and last strands, which also effect barrel closure, establish parallel hydrogen bonding. However, peptide backbones interacting as antiparallel β-sheets are structurally more stable, withstand more distortions, and establish optimal hydrogen bond lengths and bonding geometry than parallel β-sheets (19). No study has yet been carried out to address the evolutionary and structural significance of an odd-stranded mOMP. In mitochondria, VDACs are the most abundant mOMPs. They constitute ∼50% of the total protein content of the OMM and are conserved from yeast to humans. They function as metabolite flux channels that primarily carry out selective bidirectional transport of ATP/ADP, NADH/NAD^+^, nucleotides, Ca^2+^, other ions, and metabolites in a voltage-dependent manner (8, 24). Three VDAC isoforms are known in humans, with isoforms 1 and 2 being functionally indispensable for metabolite transport and mitochondria-mediated apoptosis (8). In particular, of the four mOMPs characterized thus far (9), only VDACs have been studied extensively both structurally and functionally, making this β-barrel ideally suited to deduce the energetic, functional, and phenotypic consequences of structurally re-engineering a mOMP.

Of the three human VDAC isoforms 1, 2, and 3, VDAC isoform 2 (hV2^WT^) is the only isoform that is indispensable in humans, because it binds and inactivates the Bcl2-homologous antagonist/killer, thereby performing an anti-apoptotic role (25, 26). Hence, we chose hV2^WT^ as our model β-barrel to deduce whether its unique 19-stranded β-barrel architecture is also functionally significant. We addressed this by structurally re-engineering the hV2^WT^ scaffold to 16-, 17-, 18-, 20-, and 21-stranded β-barrels. We additionally analyzed the contribution of the lipidic environment in modulating the stability of these scaffolds. For example, phosphatidylglycerol (PG) levels are negligible, and cardiolipin (CL) levels are elevated in the OMM, under physiological homeostatic conditions (27, 28). An increase in PG levels with a corresponding decline in CL content is one of the early triggers of apoptosis (27). Studies also suggest the relocation of CL to the outer leaflet of the OMM as a molecular marker of mitophagy (29). Because VDAC2 is linked directly with mitostasis and apoptosis (26), we reasoned that this barrel should display sensitivity to the lipid headgroup in the OMM, particularly to PG levels that accumulate primarily during defects in mitochondrial lipid metabolism, and alterations in the PG and CL levels during early events in mitoptosis. We provide molecular evidence that a 19-stranded β-barrel offers a unique physicochemical and functional advantage to mOMPs. We show that the 19-stranded structure is metastable, allowing VDAC2 to establish superior protein–lipid interactions and selective sensitivity toward different lipid headgroups, and is functionally the most proficient. Our findings provide an evolutionary basis for the formation, selection, and divergence of 19-stranded scaffolds exclusively in mitochondria.

## Results

### 19-Stranded structure favored in PC membranes but sensitive to lipid headgroup

**Figure 1.**
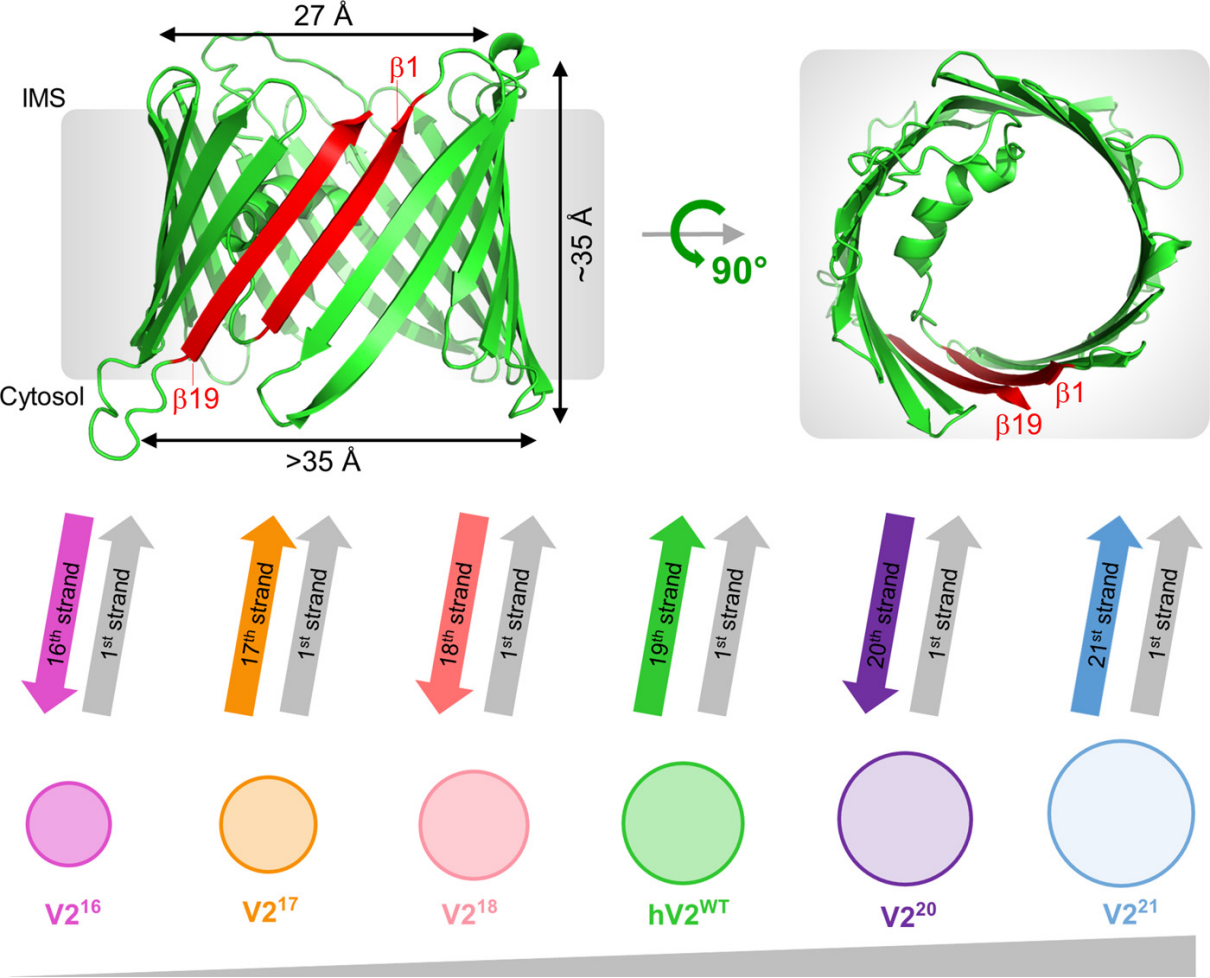
**Engineering human VDAC2 barrel variants.** *Upper panel*, I-TASSER generated structure of hV2^WT^ highlighting the transmembrane span and pore dimensions. The 1st and 19th strands (in *red*) form parallel hydrogen bonds. *Lower panel*, engineered variants of hV2^WT^ ordered schematically in increasing pore size from V2^16^ (16-stranded barrel) to V2^21^ (21-stranded barrel). The last strand that effects barrel closure with the 1st strand is indicated in each case. The color scheme and nomenclature for each variant used here is retained throughout.

First, we engineered scaffolds with odd-stranded (17, 21) or even-stranded (16, 18, 20) barrels of varying pore diameters (Fig. 1 and Fig. S1). We achieved this by strand deletion (V2^16^, V2^17^, or V2^18^) or strand addition (V2^20^ or V2^21^) at the C terminus using the 19-stranded structure as the starting scaffold. The N-terminal strand additional bears the voltage-sensor helix, and the deletion of any one strand (β2–β18) would reverse the directionality of all subsequent β-strands and modify the VDAC scaffold sizably. Therefore, the C-terminal strands were chosen specifically for deletion or duplication to ensure that the engineered barrel structures have the least perturbation in their amino acid interaction network (both hydrophobic protein–lipid and hydrophilic intrapore interactions). These strands also contain the mitochondrial targeting signal (30, 31), allowing us to examine whether this element carries an additional structural role. hV2^WT^ is highly aggregation-prone, and misfolding results in the formation of visible aggregates within minutes of initiating the folding process. We have observed previously that hV2^WT^ is stabilized under conditions of bilayer mismatch (32). Therefore, we examined the influence of the lipidic environment on the stability of hV2^WT^ and the engineered barrels using temperature-mediated barrel unfolding in PC and doped PC–PG and PC–PE membranes. All sample preparations followed rigorous procedures to check for misfolding and aggregation (see “Experimental procedures”).

**Figure 2.**
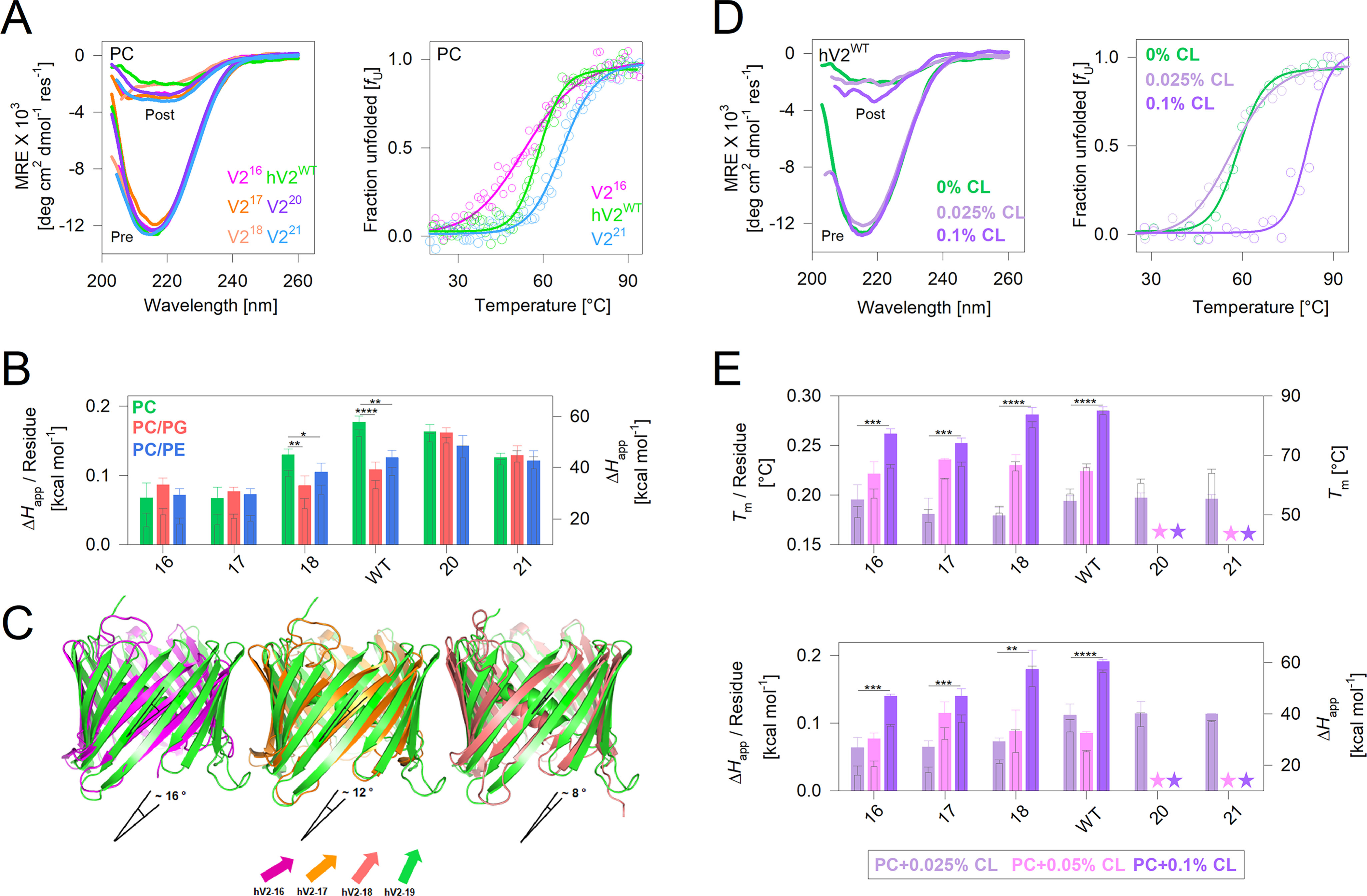
**Enthalpic contribution in 19-stranded hV2^WT^ barrel show lipid–sensitive variations.** *A*, *left panel*, representative far-UV CD profiles of folded hV2^WT^ and its engineered variants in DMPC showing comparable secondary structure content in all proteins. Profiles before (*Pre*) and after (*Post*) thermal denaturation are shown. *Right panel*, normalized representative thermal denaturation profiles monitored at 215 nm using far-UV CD (complete data including raw profiles is in Figs. S2 and S3). The data were fitted to a two-state thermal unfolding model (fits are shown as *solid lines*) to derive various thermal parameters. Note that despite variation in the absolute *T*_m_ (as seen in the profiles), the per-residue *T*_m_ does not show significant variation (Fig. S2*B* and S3*A*). *B*, change in unfolding enthalpy (Δ*H*_app_) for hV2WT and its variants compared across multiple lipid conditions. *Filled bars* (*left axis*) represent per-residue enthalpy, and *hollow bars* (*right axis*) represent total enthalpy. *Error bars* represent S.D. derived from three to five independent experiments that were analyzed independently (also see Fig. S2). *C*, comparison of change in barrel shear number because of the change in strand tilt angle, from the end structure of a 200-ns all-atom MDS. Shown is the overlay of V2^16^, V2^17^, and V2^18^ structures on hV2^WT^; the change in strand tilt angle (indicated below each structure) was calculated using PyMOL v2.0.1. *D*, representative far-UV CD (*left panel*) and thermal denaturation (*right panel*) profiles of engineered hV2^WT^ variants monitoring the effect of increasing percentage of CL (0.025–0.1% CL) doping in DMPC. *E*, comparison of the thermal parameters *T*_m_ (*top panel*) and enthalpy (*bottom panel*), measured for the engineered barrel variants in increasing percentages of CL doping. *Filled bars*, per-residue value; *hollow bars*, total value. *Error bars* represent the S.D. from two independent experiments fitted independently. *Stars* indicate values that could not be measured. The color code used is shown in the *box* below the plot. *B* and *E*, statistical analysis was carried out using a two-tailed *t* test. *, *P* ≤ 0.05; **, *P* ≤ 0.01; ***, *P* ≤ 0.002; ****, *P* ≤ 0.0004.

Unfolding of VDAC is coupled with aggregation (33). Hence, thermal denaturation measurements capture the changes associated with irreversible unfolding and aggregation of VDACs (Fig. 2*A* and Figs. S2 and S3) (32, 34). They provide two important parameters, namely (i) *T*_m_, which is the midpoint temperature wherein half the population is unfolded (Fig. S2*B*); and (ii) Δ*H*_app_, the apparent enthalpy change upon protein unfolding (Fig. 2*B*). Two other thermal parameters, namely the apparent free energy of unfolding (Δ*G*_U_) and change in entropy (Δ*S*) were also deduced (Fig. S4). Because the nature and number of noncovalent interactions established in the aggregated state of the protein cannot be estimated accurately, entropic changes during VDAC unfolding and interactions formed in the aggregated state were used for qualitative comparisons. The parameters *T*_m_ and Δ*H*_app_ are influenced by the number of interactions established in the folded barrel, which is expected to increase with protein size. Hence, we calculated the per-residue contribution to the *T*_m_ and Δ*H*_app_ and compared the results for each protein (Fig. 2, *A* and *B*, and Figs. S2 and S3, *A* and *B*). The *T*_m_ does not vary substantially from V2^16^ to V2^21^ across all lipidic conditions (Fig. S2*B*).

**Figure 3.**
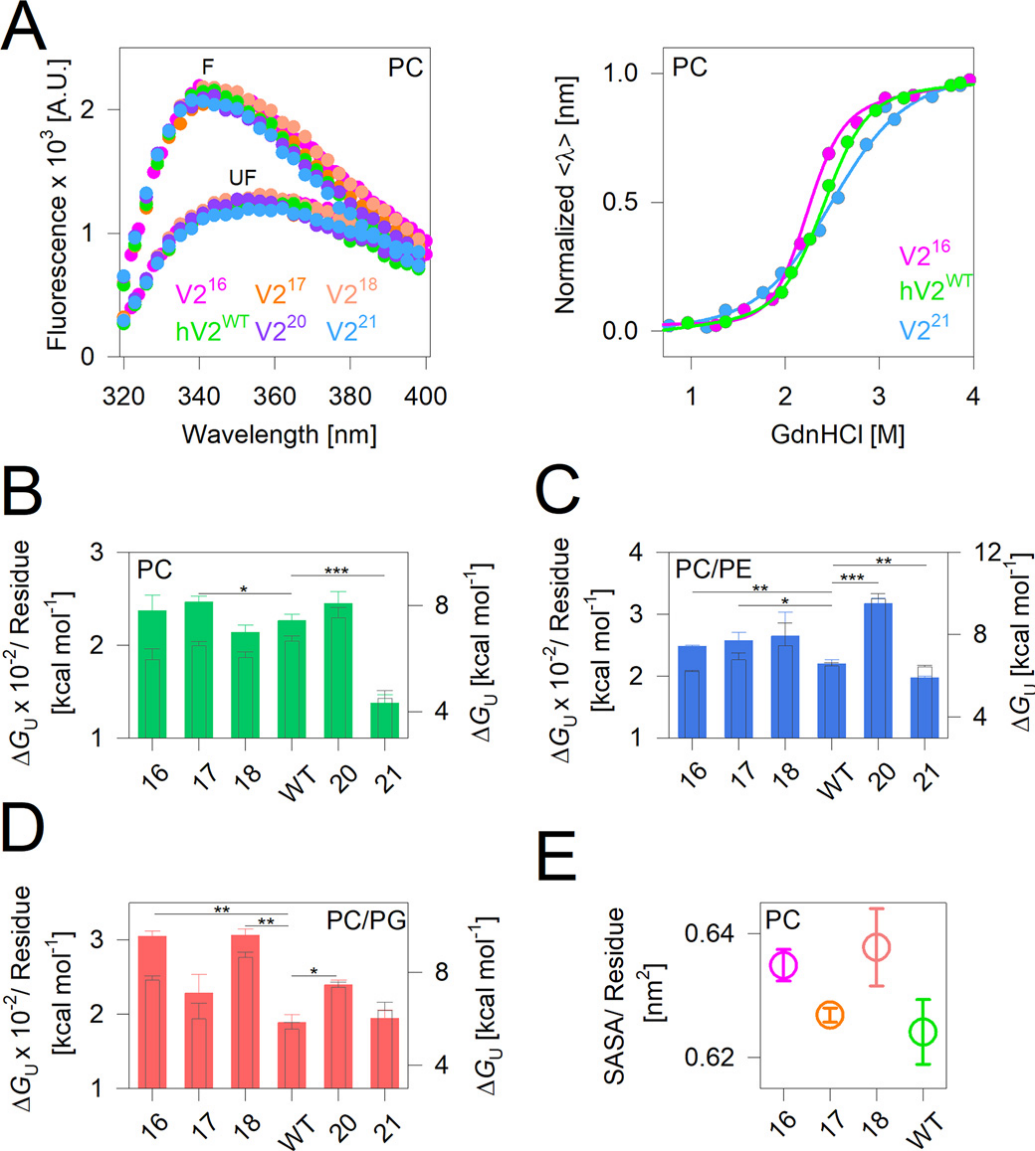
**Thermodynamic measurement show lipid-dependent lowering in hV2^WT^ stability.** *A*, *left panel*, representative tryptophan fluorescence emission profiles of the folded (*F*) and unfolded (*UF*) proteins. Upon folding, all barrel variants exhibit a comparable blue-shifted increase in tryptophan fluorescence. *Right panel*, representative two-state unfolding profiles measured as the weighted mean of the fluorescence emission intensities and normalized between 0 (folded) and 1 (unfolded). The data were fitted to the two-state folding mechanism (fits are shown as *solid lines*) to derive the thermodynamic parameters. Also see Fig. S5. *B–D*, equilibrium unfolding free energy change (Δ*G*_U_) shown as per-residue (*filled bars*) and total (*hollow bars*) values for hV2^WT^ and the engineered barrels in DMPC (*B*), and DMPC doped with DMPE (*C*) or DMPG (*D*). The *error bars* represent S.D. derived from three to five independent experiments fitted independently. *B–D*, statistical analysis was carried out using a two-tailed *t* test. *, *P* ≤ 0.05; **, *P* ≤ 0.01; ***, *P* ≤ 0.002). Also see Fig. S5*B*. *E*, average SASA derived for the transmembrane segment from two independent 200-ns simulations for V2^16^–V2^18^ and hV2^WT^. Simulations could not be carried out on V2^20^ and V2^21^ (see “Experimental procedures” for details).

**Figure 4.**
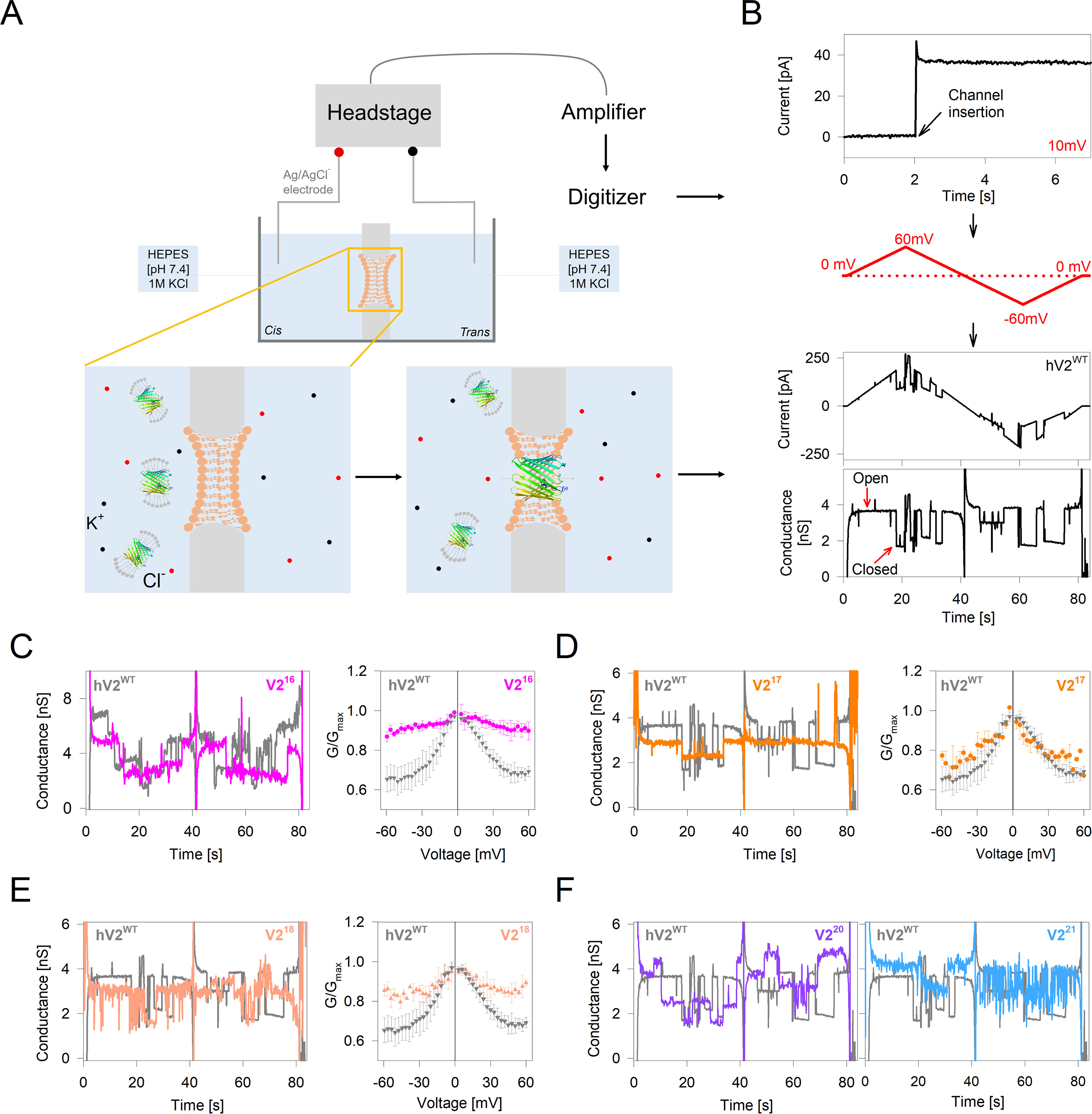
**Gating behavior of engineered barrel variants is suboptimal.** *A*, schematic representation of the experimental setup for electrophysiology measurements in a DiPhPC planar bilayer membrane painted across a 150-μm aperture. *B*, *top* to *bottom*, representative data showing single-channel insertion at a holding voltage of 10 mV for hV2^WT^. A triangular voltage ramp program (0 to ±60 mV) is applied across the membrane to record the voltage-dependent change in ion flux for the channel. The measured current (in pA) and corresponding conductance (in nS) shows a drop beyond ±30 mV, indicating the transition of the open channel to its subconductance state. *C–E*, *left panels*, representative single-channel conductance of V2^16^ (*C*; two channels), V2^17^ (*D*), and V2^18^ (*E*) overlaid with hV2^WT^ (in *gray*). *Right panels*, comparison of *G*/*G*_max_ from multichannel voltage ramp measurements (20–100 channels). Errors were derived from two to four independent experiments. Several multichannel recordings produced nonresponsive channels in the cyclic voltage ramping for V2^16–18^ and were not used in the analysis. *F*, representative single-channel conductance of V2^20^ (*left panel*) and V2^21^ (*right panel*) overlaid with hV2^WT^ (in *gray*). Note that V2^20–21^ destabilized the DiPhPC bilayer, and therefore, recordings with good signal-to-noise are limited for these constructs.

Interestingly, the per-residue enthalpic contribution, indicative of intraprotein and protein–lipid interaction strength, is higher for the 19–21 stranded structures (Fig. 2*B* and Fig. S2, *C–E*). In particular, the enthalpic contribution is highest for the 19-stranded hV2^WT^ in PC membranes and is lowered nearly 1.5–fold upon PG or PE doping. Hence, hV2^WT^ structure is sensitive to the lipid headgroup in the membrane. V2^20^, although retaining a high enthalpy, does not exhibit a similar lipid-dependent variation (Fig. 2*B*). The per-residue apparent Δ*G*_U_ (Fig. S4*B*) and Δ*S* (Fig. S4*C*) measured from thermal denaturation are in good agreement with our conclusions from the Δ*H*_app_. To understand the source of this enthalpic variation, we generated the engineered structures of V2^16^–V2^18^ from hV2^WT^ *in silico* (V2^20^ and V2^21^ could not be generated; see “Experimental procedures” for details). Our all-atom molecular dynamics simulations (MDSs) reveal that in V2^16^–V2^18^, the strand arrangement with respect to the bilayer axis is altered (Fig. 2*C*; also see Fig. S6*A* and Movies SM1–SM3). Here, the strand tilt angle decreases progressively from V2^16^ to hV2^WT^ and could be lower in V2^20^ and V2^21^. A decrease in strand tilt concomitantly lowers the shear number. The latter alters the hydrogen-bonding pattern in the transmembrane region of these β-barrels. We surmise based on the simulations that changes in the number and strength of the H-bonds could contribute significantly to the measured change in enthalpy across these engineered barrels, despite the membrane composition.

Another interesting finding from the thermal measurements is how the lipid composition selectively modulates hV2^WT^ inter- and intramolecular interaction, thereby influencing the enthalpic stabilization. Under physiological conditions, the outer mitochondrial membrane is enriched with PC (>50% w/w of the total lipid) and PE (∼30% w/w). Healthy mitochondria lack PG lipids in their OMM, because PG is converted metabolically to CL (23). Therefore, we assessed the influence of CL doping (0.025%-0.1%) on the thermal stability of hV2^WT^ and the engineered V2^16-21^ variants (Fig. 2, *D* and *E*, and Figs. S2*A* and S3*C*). An increase in both the *T*_m_ (Fig. 2*E*, *top panel*) and enthalpy (Fig. 2*E*, *bottom panel*) suggests that an increase in CL concomitantly increases the stability of all proteins in proportion to the accessible surface available to the lipid membrane. It is likely that CL nonspecifically interacts with hV2^WT^ and the V2 variants, stabilizing the scaffold by forming better protein–lipid interactions. A corollary to this finding is that lowering of CL in the OMM caused by metabolic defects in CL biosynthesis could concomitantly lower hV2^WT^ stability.

Comparing our observations across the various lipids allows us to infer that hV2^WT^ is the only barrel structure that is most sensitive to the PC, PE, and PG levels in the membrane. We find that hV2^WT^ stability is lowered enthalpically by ∼1.5–fold in the presence of PE or PG, when compared with PC membranes. The 19-stranded structure possesses an optimal strand tilt and shear number, which allows hV2^WT^ respond to changes in the lipid microenvironment by altering the scaffold stability. An increase in PG levels and a corresponding lowering of CL in the OMM during apoptosis is known to trigger VDAC oligomerization (27). Considering the specific role of human VDAC2 in apoptosis, a functional switch in the protein function based on cellular physiology might indeed be facilitated chiefly by a 19-stranded β-barrel structure.

### Thermodynamic stability of 19-stranded structure lowered in nonlamellar membranes

Using equilibrium thermodynamic measurements, we measured the change in the Gibbs free energy of unfolding (${\Delta G}_{U}^{0,H2O}$; Δ*G*_eq_) for hV2^WT^ and the engineered barrels. Here, protein unfolding was achieved using guanidine hydrochloride as the chemical perturbant and by incubating the reaction at 25 °C, until equilibrium was achieved in 24 h. The unfolding was monitored using a change in the fluorescence emission profiles of the four intrinsic (interface) tryptophan residues. Unlike thermal unfolding (shown in Fig. 2), chemical denaturation is reversible. Here, hV2^WT^ and its variants exhibit a two-state unfolding transition between the folded and unfolded states without a detectable intermediate (Fig. 3*A* and Fig. S5*A*). The Δ*G*_eq_ thus derived was converted to a per-residue contribution, and the global change in stability was compared in PC and in doped PE or PG lipids (Fig. 3, *B–D*, and Fig. S5*B*).

**Figure 5.**
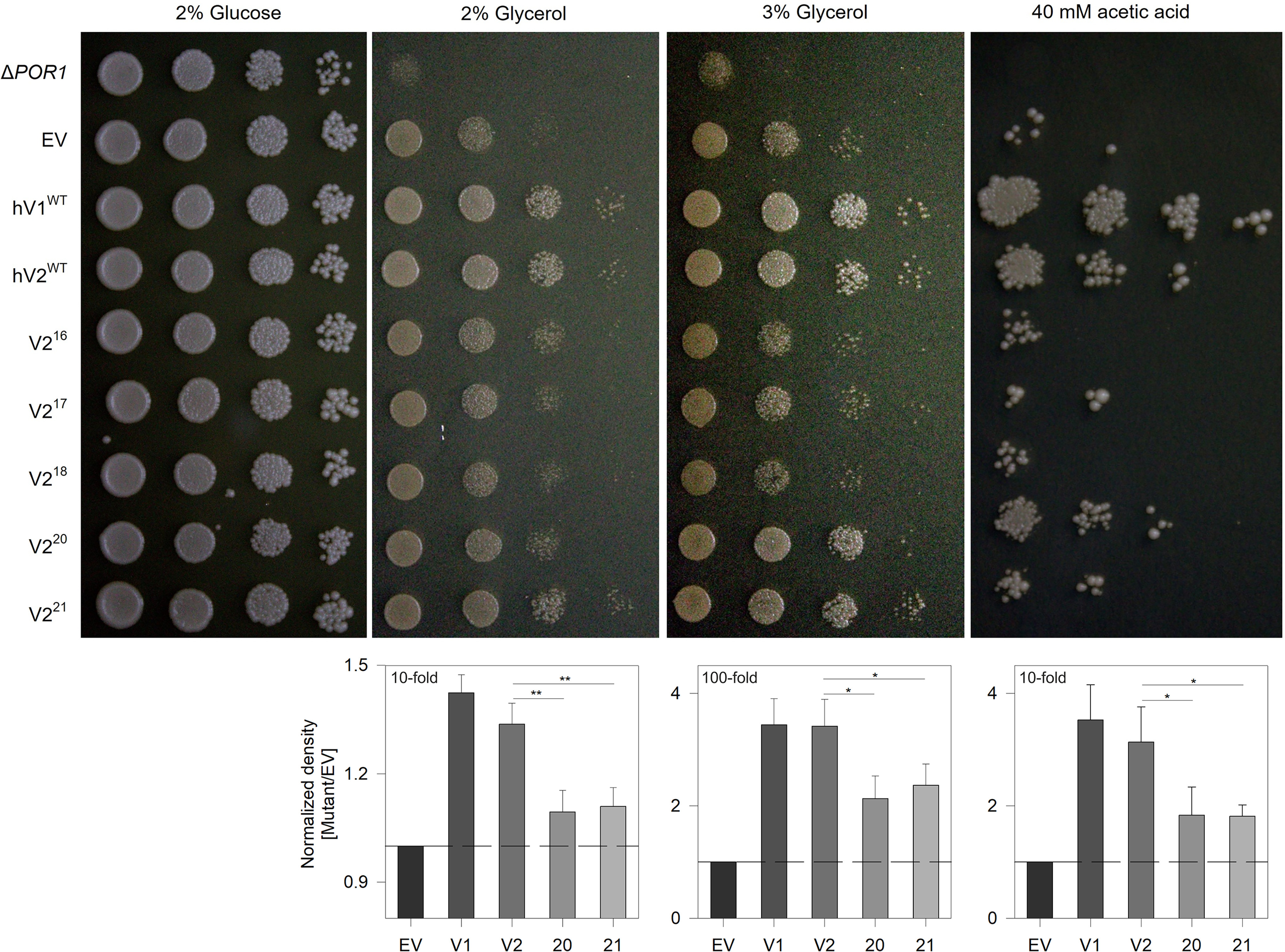
**Complementation with hV2^WT^ supports yeast survival under restrictive growth conditions.** Serial spot dilution assay to measure survival efficiency of *POR1*-deficient yeast. Δ*POR1* yeast strain BY4742 (*por1*Δ) transformed with pYX212 shuttle vector (*EV*) carrying WT human VDAC1 (hV1^WT^), human VDAC2 (hV2^WT^), and the engineered barrels (V2^16^–V2^21^) were spotted (*left* to *right*, fold dilution = 0, 10, 100, and 1000) in medium containing glucose or glycerol (nutrient restriction) as the sole carbon source. The survivability of yeast expressing the VDAC variants was assessed on two glycerol concentrations, namely, 2 and 3%. The cells were also grown in glucose-containing medium additionally containing acetic acid to induce oxidative stress. Quantification obtained from spot density (2% glycerol and acetic acid, 10-fold; 3% glycerol, 100-fold) from >12 independent experiments is presented as histograms (*lower panels*). EV has a likely growth advantage over *por1*Δ, because of the additional expression of the URA3 gene (selection marker for pYX212). Therefore normalization was done in each case against the growth of EV. Statistical analysis was carried out using a one-tailed *t* test. *, *P* ≤ 0.05; **, *P* ≤ 0.01. Note how yeast expressing strand deletion constructs show poor survival, whereas the growth of cells expressing V2^20^ or V2^21^ is less compared with hV1^WT^ and hV2^WT^.

Unlike thermal unfolding measurements, we find from the Δ*G*_eq_ that hV2^WT^ shows selective sensitivity to its lipidic environment. The presence of PE or PG lowers the enthalpy of hV2^WT^ unfolding and aggregation, when compared with PC (Fig. 2). However, comparison of the Δ*G*_eq_ across the three different lipidic conditions reveals that hV2^WT^ exhibits selective destabilization only upon addition of PG (Fig. 3, *B–D*, and Fig. S5*B*). Additionally, the hV2^WT^ energetics derived from both approaches provides a direct readout of how PG invariably modulates hV2^WT^ stability. Unlike PC and PE, which constitute >50% and ∼30% of the OMM under physiological conditions, PG levels are negligible (23). We speculate that an increase in PG with concomitant destabilization of VDACs could signal mitochondrial stress and the activation of metabolic pathways for CL biosynthesis (23).

Next, we examined the effect of PC, as well as PE and PG doping, on the engineered V2 variants. The stability of V2^21^ is low in all three conditions, particularly in PC. The addition of PE in PC membranes increases the stability of nearly all the engineered V2 barrels, particularly when compared with the Δ*G*_eq_ of hV2^WT^ in the same condition (Fig. 3*C*). The most interesting outcome from changes in Δ*G*_eq_ is seen upon doping PC membranes with PG (Fig. 3*D*). Here, both the engineered even-stranded barrels (wherein barrel closure is achieved through antiparallel H-bonds) and odd-stranded barrels (wherein barrel closure is achieved through parallel H-bonds) show distinct differences from hV2^WT^. The measured per-residue Δ*G*_eq_ is higher for V2^16^, V2^18^, and V2^20^ compared with hV2^WT^. This observation is the combined effect of increase in stability of the V2 variants and the decrease in stability of hV2^WT^. Our results can be explained to a great extent by the stronger H-bonding established by the first and last strands of the even-stranded barrels, which are less accessible to solvation and perturbation. In contrast, the odd-stranded barrels V2^17^, hV2^WT^, and V2^21^ show nearly similar Δ*G*_eq_ in PG (Fig. 3*D*). Moreover, the Δ*G*_eq_ of V2^21^ is higher in both PC/PE and PC/PG compared with PC (compare Fig. 3, *B–D*). When compared across the three lipid conditions, the only β-barrel that undergoes destabilization and shows a decrease in Δ*G*_eq_ upon addition of PG is hV2^WT^ (Fig. 3*D*, and Fig. S5*B*).

Although the involvement of VDACs in the formation of the permeability transition pore has been debated (35), studies also indicate that adverse changes in the lipid profile of the OMM could trigger the observed VDAC oligomerization and release of mitochondrial DNA (36). The most significant change in the lipidome is the lowering of CL and a concomitant increase in PG content (23, 27, 28). We also infer from our equilibrium measurements (Fig. 3, *B–D*), that the hV2^WT^ structure is intrinsically metastable. Hence, protein–lipid interplay plays a vital role in homo- and heterooligomerization of VDACs in the OMM under physiological conditions, and further destabilization of VDACs by PG could trigger the switch to mitoptosis. Our finding that the sensitivity of the β-barrel to changes in the lipidic milieu is highest for the 19-stranded structure offers a physicochemical basis for the evolutionary selection of this odd-stranded barrel.

To validate our interpretation, we performed *in silico* studies (all-atom MDS) for V2^16^–V2^18^ and hV2^WT^. We calculated the solvent-accessible surface area (SASA) per residue, root-mean-square fluctuation (RMSF), and radius of gyration (*R*_g_). With increase in barrel size, we obtain a change in SASA (Fig. 3*E*), *R*_g_, and RMSF (Fig. S6). High SASA and *R*_g_ coupled with low RMSF, which together represent structural compaction of the protein caused by stronger intraprotein interactions, are seen largely for V2^16^ and V2^18^. In contrast, we observe generally lower values for SASA, which is accompanied by an increase in RMSF and is coupled with no substantial increase in *R*_g_ (despite increase in strand number) for the odd-stranded structures of V2^17^ and hV2^WT^ (details in Fig. S6). These observations together signify highly dynamic structures with lowered barrel compaction for V2^17^ and hV2^WT^ and indicate better protein–lipid interactions. Putting together our findings from the MDS with the thermal (Δ*H*_app_) and equilibrium (Δ*G*_eq_) stability measured for hV2^WT^ provides an energetics perspective on how a 19-stranded structurally dynamic barrel adapts in the OMM. Lowered solvation, higher lipid–protein interaction, and the destabilization introduced by parallel hydrogen bonding can together account for a highly lipid-modulated channel that is better adapted for protein oligomerization and its functional relevance in nonlamellar membranes of mitochondria. Our findings are also in excellent agreement with previous measurements of high hV2^WT^ stability arising from stronger barrel–micelle interaction (37, 38).

### Strand number modification impairs voltage gating and cell survival

Protein structures with moderate stability, *i.e.* metastable proteins, are evolutionarily optimized largely in favor of their *in vivo* function (39, 40). Therefore, our finding that hV2^WT^ adopts a metastable structure could bear functional significance. VDACs are metabolite flux transporters in the OMM that show gating characteristics sensitive to the membrane potential. The resting membrane potential across the OMM is largely ∼0 mV and varies up to 40 mV under conditions of cellular homeostasis (41–43) and is altered during mitochondrial stress. In 1 m KCl, VDACs retain an open anion-selective state with a conductance of ∼4 nS, and an increase in the membrane potential beyond ± 30 mV causes the channel to switch to a cation–selective subconductance state of ∼2 nS (38, 44). We studied the voltage-gating property of hV2^WT^ and the engineered β-barrels using single-channel and multichannel electrophysiology measurements (Fig. 4, *A* and *B*).

Representative single-channel conductance profiles of hV2^WT^ and the engineered barrels across a voltage ramp of ± 60 mV is presented in Fig. 4 (*C–F*). Comparison of the single conductance shows a variation in proportion to the barrel size, with decrease in conductance as the pore size decreases and an increase in conductance upon strand addition. For example, we obtain an average open channel conductance of ∼3.5 nS for hV2^WT^, which is reduced to ∼3.2 nS for V2^18^, ∼2.8 nS for V2^17^, and ∼2.5 nS for V2^16^. On the other hand, the conductance is increased to ∼4.0 nS for V2^20^ and ∼4.3 nS for V2^21^ (Fig. 4, *C–F*). Interestingly, we observe nonlinear differences when comparing the *G*/*G*_max_ values for multichannel data (Fig. 4, *C–E*). Here, the voltage-dependent gating characteristic is substantially compromised in the even-stranded barrels V2^16^ and V2^18^ and is lowered marginally for V2^17^. Additionally, V2^16–18^ form multichannels that are noisier or respond poorly to the applied voltage (Fig. 4, *C* and *E*). In the case of V2^20^ and V2^21^, although the channels responded to the applied voltage (Fig. 4*F*), the insertion of multiple channels caused significant destabilization of the membrane. Put together, the voltage-dependent multichannel gating characteristics of hV2^WT^ is superior, suggesting that the 19-stranded structure is functionally apposite. We also find that the engineered barrels display gating characteristics (albeit compromised), suggesting that the voltage-gating sensor in VDAC is not specific to the C-terminal strands. Our observation that ∼100 channels of hV2^WT^ together respond efficiently and synchronously to the membrane potential without perturbing the bilayer membrane allows us to propose that the 19-stranded β-barrel was selected for its superior gating characteristics and voltage-regulated conductance.

To further probe the functional importance of a 19-stranded VDAC structure, we carried out complementation assays in *POR1*-depleted yeast (VDAC in yeast is termed porin) (4, 5, 17). The metabolism of nonfermentable carbon sources by yeast requires mitochondria containing a functional porin in the outer membrane. For example, *POR1*–depleted yeast survive in media containing fermentable sugars such as glucose (2% YPD (yeast, peptone, and dextrose)), However, these cells exhibit substantial growth deprivation followed by cell death when glycerol is the sole carbon source (2 and 3% YPY (yeast, peptone, and glycerol)). To check for *in vivo* function of the engineered barrels, we assayed the survival of *por1*Δ yeast transformed with the engineered strand variants. Yeast cells transformed with human VDAC1 (hV1^WT^) served as our reference in the serial dilution assays.

The results of our yeast survival assays are illustrated in Fig. 5. The survival efficiency of yeast expressing hV1^WT^ and hV2^WT^ are comparable. However, cells expressing V2^16^–V2^18^ show poor growth when compared with EV (Fig. 5), although these variants form voltage-gated channels *in vitro* (Fig. 4), suggesting poor mitochondrial targeting of hV2^16–18^ after removal of the 19th strand (30, 45). This observation also signifies the importance of β19 in mitochondrial targeting of VDAC. Therefore, we limited our comparisons to hV1/2^WT^ and V2^20–21^. Interestingly, cells expressing V2^20^ and V2^21^ also show poor growth compared with hV2^19^, although the mitochondrial targeting signal is retained in these variants (Fig. 5, *lower panels*). Hence, the engineered V2^20–21^ variants are functionally less efficient than hV1^WT^ and hV2^WT^ *in vivo*.

We confirmed our findings further by subjecting yeast cells to oxidative stress. Here, redox stress increases the local concentrations of peroxide and superoxide radicals in the mitochondrial compartments. Because VDACs can conduct free radicals in their open state, superior channel gating characteristics and rapid structural conversion to the cation-selective subconductance state (44) will prevent oxidative damage of cytosolic proteins and increase cell survival. Our findings reveal that yeast survival efficiency follows the order hV1/2^WT^ > V2^20^ ≈ V2^21^ (Fig. 5), supporting our electrophysiology measurements that (i) the engineered 20- and 21-stranded barrels are functionally compromised compared with the 19-stranded scaffold, and (ii) cell survival under redox stress relies on efficient voltage-gated switch of the 19-stranded VDACs to the subconductance state. Our findings support the functional superiority of the 19-stranded β-barrel in voltage gating and regulating mitochondrial bioenergetics.

## Discussion

All known bacterial outer membrane proteins are even-stranded β-barrels that reside in membranes rich in phosphatidylethanolamine (PE; ∼70–80%) and phosphatidylglycerol (PG; ∼20–30%). In interesting contrast is the mitochondrial outer membrane, which is different from its prokaryotic ancestor in (i) lipidic composition, wherein mitochondrial membranes are enriched with phosphatidylcholine (PC; >50%) with ∼30% PE, ∼15% phosphatidylinositol, ≤ 1% cardiolipin (CL), and 0% PG (22, 23); and (ii) possessing at least three abundant outer membrane protein β-barrels, namely VDACs, Tom40, and Mdm10 (in yeast) that adopt odd-stranded, *i.e.* 19-stranded β-barrel structures (9). Being the most abundant protein and constituting 50% of the OMM protein content, VDACs dictate both mitochondrial biogenesis and mitochondrial bioenergetics. Hence, the functional significance of its unique 19-stranded scaffold (*versus* an 18- or 20-stranded scaffold), and the concept of an odd-stranded barrel is a fundamental question that warrants comprehensive analysis.

Our findings reveal that the 19-stranded β-barrel is functionally the most competent for metabolite flux across the OMM and additionally is the only scaffold that exhibits a lipid-dependent alteration in its stability. We show here that a 19-stranded metastable structure is generic to the OMM and is fundamental for mitoptosis. For example, although V2^20^ possesses both an enthalpic and energetic advantage, this scaffold is less sensitive to an increase in the PG headgroup. Indeed, lipid headgroups have a major impact on both stability and function of several membrane proteins including VDAC (27). In the OMM, the 19-stranded structure is sensitive to dynamic changes in the lipid composition under physiological conditions and disease states. Hence, the mitochondrial membrane lipids can optimally regulate the function of only the 19-stranded structure, whereas the engineered scaffolds are largely unaffected or exhibit an increase in stability in PC–PG lipids.

We find that modulation of VDAC structure for physiological function, enthalpically optimal protein–lipid interactions, and response to changes in the OMM microenvironment is possible only with the 19-stranded scaffold. Moreover, the per-residue contribution to enthalpy and free energy does not correlate with the barrel size or exhibit a dependence on the H-bond pattern (parallel H-bonding is ideally weaker than antiparallel H-bonding). A closer examination of the MDS data reveals how even-stranded barrels are structurally rigid and less dynamic, thereby increasing solvent accessibility within the barrel pore, allowing water molecules to perturb interstrand H-bonds. In contrast, the odd-stranded barrels are more structurally dynamic, establish better protein–lipid interactions, and display a lower surface area for solvent-mediated perturbation. Enthalpic contributions from protein–lipid interactions, when considered with optimal shear number, reveal the biophysical advantage of a 19-stranded scaffold. In contrast, the engineered barrels are devoid of at least one of these characteristics and explain why these structures would be both thermodynamically and functionally deselected in evolution.

Bacterial β-barrels are presented with hostile extracellular environments and a distinctly asymmetric lipopolysaccharide–phospholipid membrane (46). These unusually rigid scaffolds accommodate prolonged turnover times through significantly high kinetic stability (13, 47). In contrast, VDACs require a metastable scaffold to achieve structural dynamicity necessary for its homo- and heterooligomerization and diverse functions ranging from flux of various metabolites, signaling in the asymmetric mitochondrial bilayer membrane, to its dynamic interactome with metabolic, cytosolic, and apoptotic proteins (5, 25, 48). We propose that the evolutionary need for a versatile mitochondrial channel that adapts proficiently to a membrane with a highly fluidic composition could be met only by a 19-stranded structure. We envision that in the cell, these 19-stranded β-barrels sample alternate microstates that allow VDACs, particularly isoform 2, to carry out diverse functions in the outer mitochondrial membrane.

Although it is interesting to speculate that Tom40, the highly conserved 19-stranded mitochondrial protein translocase, shared an evolutionary selection pressure similar to that of VDACs, what still remains to be identified is the ancestral sequence that gave rise to these unique protein scaffolds with divergent functions. Coevolution analysis has indeed predicted the likely existence of a phylogenetic history between these two vital mitochondrial channels (17). Whether the 19-stranded Tom40 also exhibits an energetically optimized metastable structure, as we find here for VDAC, remains to be studied. It is also of interest to examine whether structural alterations in the 19-stranded VDAC β-barrel occur during PE build-up, which triggers the formation of oligomeric VDAC structures that culminate in OMM permeabilization and cytochrome *c* release (48). The challenges ahead include the development of methods for detecting such subtle conformational changes in these proteins in the mitochondrial membrane that have irrevocable consequences on the fate of the cell.

## Experimental procedures

### Cloning, scaffold design, and mutant generation

All the constructs used in this study, namely WT human *VDAC2* gene, as well as all the strand deletion and addition mutants, were cloned in pET3b. Genes for the 18-, 17-, and 16-stranded barrels were generated by introducing stop codons at residues 283, 264, and 252, respectively, using site-directed mutagenesis of the pET3b-*VDAC2* template. For the 20- and 21-stranded barrels, the 18th strand or 18th and 19th strands, respectively, were duplicated after the last residue and before the stop codon using overlapping PCR. All proteins listed in Fig. 1 were expressed tag-less as inclusion bodies in *Escherichia coli* BL21(DE3) cells and purified by anion exchange chromatography (Fig. S1), as explained in previous reports (32, 37).

### Lipids and detergents

All lipids and detergents were purchased from Avanti Polar Lipids, Inc. (Alabaster, AL, USA). Bicelles were prepared with the short-chain 12-C detergent, *n*-dodecylphosphocholine (DPC) and the long-chain lipid 1,2-dimyristoyl-*sn*-glycero-3-phosphocholine (*di*C14:0 PC, DMPC, PC). Doped bicelles were prepared by incorporating 20% of 1,2-dimyristoyl-*sn*-glycero-3-phospho-(1´-rac-glycerol) (*di*C14:0 PG, DMPG, PG), or 1,2-dimyristoyl-*sn*-glycero-3-phosphoethanolamine (*di*C14:0 PE, DMPE, PE) and increasing percentages (0.025, 0.05, and 0.1) of CL (heart, bovine) in DMPC:DPC bicelles. 1,2-Diphytanoyl-*sn*-glycero-3-phosphocholine (DiPhPC) was used to form planar lipid bilayers for single-channel electrophysiology experiments in black lipid membranes.

### Bicelle preparation

Dried films of DPC, DMPC, or doped DMPC were resuspended in the desired concentrations in 100 mm NaCl and 50 mm phosphate buffer (pH 7.2) by heating at 42 °C and intermittent vortexing cycles, until the solution became homogenous. Isotropic bicelles of *q* = 0.5 were prepared by mixing a fixed concentration of resuspended DPC (final concentration, 20 mm) and DMPC/DMPG/DMPE/CL (final concentration, 10 mm) in the solution containing 50 mm sodium phosphate buffer (pH 7.2) and 100 mm NaCl additionally supplemented with 10 mm DTT. This bicelle mix was subjected to three to five cycles of heating (42 °C) and cooling on ice with intermittent vortexing, until the solution turned viscous at 42 °C. Homogeneity of select bicelle preparations was verified by measuring the dissociation enthalpy profile using differential scanning microcalorimetry, following reported methods (32). For doped bicelle preparations, dried films of PC premixed with PG, PE or CL were used. Doped bicelles were prepared using the same procedure but with 15 heating–cooling cycles. Cooling was achieved using liquid N_2_ in 0.1% CL to ensure homogenous distribution of CL. The list of lipidic conditions used in this study is provided in Table 1.

**Table 1:** **Lipidic bicelle compositions used in this study**

Bicelle	Composition
PC	DMPC/DPC
PC/PG	(80% DMPC + 20% DMPG)/DPC*^a^*
PC/PE	(80% DMPC + 20% DMPE)/DPC*^a^*
0.025% CL	(DMPC + 0.025% CL)/DPC*^b^*
0.05% CL	(DMPC + 0.05% CL)/DPC*^b^*
0.1% CL	(DMPC + 0.1% CL)/DPC*^b^*

*^a^* The percentage of doping of long-chain lipids DMPG or DMPE in DMPC calculated based on molar ratio (the mentioned percentage is the percentage of the final molar amount of the total long-chain lipid).

*^b^* The percentage of CL is added based on a w/v calculation.

### Folding of hV2^WT^ and V2 variants in preformed bicelles

Protein folding was initiated by rapidly diluting 250 μm of the unfolded protein prepared in 8 m guanidine HCl and 10 mm DTT into preformed bicelles of *q* = 0.5. A 10-fold dilution of the protein in the folding mixture provided final concentrations of 10 mm DMPC (+DMPE/DMPG/CL), 20 mm DPC, and 25 μm protein in 50 mm sodium phosphate buffer (pH 7.2), 10 mm DTT, and 100 mm NaCl. This folding mix was subjected to three cycles of heating at 35 °C for 30 s with intermittent vortexing and then cooling on ice for 30 s, followed by overnight incubation at 4 °C with constant mixing by rotation at 15 rpm. Each folded sample was diluted 5-fold and subjected to a 1-h high-speed centrifugation at 16,900 × *g* at 4 °C to remove trace amounts of unfolded or aggregated species. The samples were checked additionally for the absence of soluble aggregates by monitoring absorbance (scattering) at 320 nm (*A*_320_ = 0) using a quartz cuvette of 1-cm path length. Folded samples were quantified using absorbance at 280 nm, and a molar extinction coefficient of 36,900 m^−1^ cm^−1^ (35410 m^−1^ cm^−1^ for V2^16^) was used. All preparations were used within 1 h of quantification.

### Thermal denaturation measurements

Folded hV2^WT^ and its engineered mutants were subjected to thermal denaturation from 4 °C to 95 °C at a ramp rate of 1 °C/min on a JASCO J-815 CD spectropolarimeter equipped with a water-cooled Peltier (32, 34). A final protein concentration of 5 μm and a lipid–protein ratio of 400:1 were used. Prior to the start of the melting experiment, wavelength scans were acquired at 4 °C using a quartz cuvette of 1-mm path length and acquisition settings of 0.5-nm data pitch, 100-nm/min scan speed, 1-s data integration time, and 1-nm bandwidth. Each data set was averaged over three accumulations and corrected for both buffer and lipid contributions. The variable temperature program was used for thermal denaturation measurements, with a ramping of 1 °C steps from 4 to 95 °C, 1-nm bandwidth, and 1-s integration time. Thermal denaturation measurements were monitored using a 1-mm path length quartz cuvette at a wavelength of 215 nm (corresponding to high β-sheet contribution to the ellipticity). The data were acquired additionally at 222 nm for verification. The data were smoothed using the means-movement method and converted to per-residue molar ellipticity (ME_215_). The fraction unfolded (*f*_U_) was calculated using Equation 1.

(Eq. 1) $$f_{\text{U}}=\left(\theta_{\text{Obs}}-\theta_{\text{F}}\right)/\left(\theta_{\text{UF}}-\theta_{\text{F}}\right)$$

Here, θ_Obs_ is the observed ME_215_ at a given temperature, and θ_F_ and θ_UF_ are the ME_215_ values for the folded protein at 4 °C and unfolded protein at 95 °C, respectively. The *f*_U_ data were fitted to a two-state thermal unfolding model (49) using Equation 2.

(Eq. 2) $$f_{U}=\frac{\left(\left(m_{F}\;T_{x}\right)\;+\;y_{F}\right)\;+\;\left(\left(\left(m_{U\;}T_{x}\right)\;+\;{y_{U}}_{\;}\right)\exp\left(-\left(\frac{{\Delta H}_{app}}{R}\right)\left(\frac{1}{T_{x}\;+\;273.15}\;-\;\frac{1}{T_{m}\;+\;273.15}\right)\right)\right)}{1\;+\;\exp\left(-\;\left(\frac{{\Delta H}_{app}}{R}\right)\left(\frac{1}{T_{x}\;+\;273.15}\;-\;\frac{1}{T_{m\;}+\;273.15}\right)\right)}$$

Here, *y*_F_ and *y*_U_ are the intercepts of the pre- and post-transition baselines, respectively. Similarly, *m*_F_ and *m*_U_ are the slopes of pre- and post-transition baselines, respectively. *T*_x_ is applied temperature that was varied from 4 to 95 °C at 1 °C increments. *R* is the gas constant (1.987 × 10^−3^ kcal K^−1^ mol^−1^). The *T*_m_ (the midpoint of thermal denaturation) and Δ*H*_app_ (apparent cooperativity of the unfolding transition) derived herein were converted to per-residue contributions. The values obtained from three to five independent experiments were averaged to obtain the mean *T*_m_ and Δ*H*_app_ and the standard deviation.

### Equilibrium unfolding measurements

hV2^WT^ and its mutants were folded in DMPC/DPC bicelles (with(out) PE or PG) and subjected to chemical denaturation using increasing concentrations of guanidine HCl (guanidine hydrochloride) from 0.16 to 6.56 m (38). Here, the final protein concentration of 5 μm and lipid–protein ratio of 400:1 were maintained for all the constructs. The reaction was set in a 96-well black microplate and monitored by following the change in tryptophan fluorescence, using an excitation wavelength of 295 nm, and emission scans were collected between 320 and 400 nm with a step size of 1 nm (top read) on a SpectraMax M5 multimode reader (Molecular Devices, Inc.). All recordings were carried out at 25 °C. Equilibrium was achieved within ∼24 h, and the 24-h data were used for the calculations. The data were corrected for buffer, lipid, and guanidine HCl contributions, and the average wavelength (<λ>) was calculated using Equation 3 (50).

(Eq. 3) $$<\lambda >\;=\;\frac{\sum_{i=0}^{n}\left(I_{i\;}\lambda_{i}\right)}{\sum_{i=0}^{n}\left(I_{i}\right)}$$

Here, *I*_i_ is the emission intensity at the wavelength λ_i_; λ_0_ = 320 nm; and λ_n =_ 400 nm. The data were fitted to a two-state linear extrapolation model using Equations 4 and 5 (50).

(Eq. 4) $$<\lambda >\;=\;\frac{\left(\left(m_{F}[D]\right)\;+{\;y}_{F}\right)+\;\frac{1}{Q_{R}}\left(\left(\left(m_{U}[D]\right)\;+\;y_{U}\right)\;exp\left(-\frac{\;\left({\Delta G}^{0,\;H2O}\right)\;+\;m[D]}{RT}\right)\right)}{1\;+\;\frac{1}{\;Q_{R}}exp\left(-\;\frac{\left({\Delta G}^{0,\;H2O}\right)\;+\;m[D]}{RT}\right)}$$

(Eq. 5) $$Q_{\text{R}}\;=\;\frac{\Sigma I_{i\left(\text{F}\right)}}{\Sigma I_{i\left(\text{U}\right)}}$$

Here, [D] represents the denaturant (guanidine HCl) concentration; *y*_F_ and *m*_F_ represent the pretransition intercept and slope, respectively; and *y*_U_ and *m*_U_ represent the post-transition intercept and slope, respectively. The cooperativity of (un)folding is represented by *m*, which is also a measure of the change in SASA upon (un)folding. *R* is the gas constant, and *T* is the temperature in kelvin. *Q*_R_ represents the normalization ratio, *i.e.* the sum of intensities (*I*_i_) of native (N) and unfolded (U) states calculated using Equation 5, as reported earlier (50). The change in Gibbs free energy of unfolding (${\Delta G}_{U}^{0,H2O}$; Δ*G*_eq_) thus derived was averaged over two or three independent experiments and compared across hV2^WT^ and its engineered variants.

### Measurement of single-channel voltage-gating characteristics

Voltage-gating property of hV2^WT^ and the engineered strand constructs were characterized using the black lipid membrane system equipped with an 8-pole Bessel filter (Warner Instruments, Harvard Apparatus Inc.). 250 μm of the unfolded protein stock prepared in 6 m guanidine HCl and 10 mm DTT was diluted 10-fold in LDAO (lauryldimethylamine oxide) micelles to achieve final concentrations of 25 μm protein and 65 mm LDAO, in 50 mm sodium phosphate (pH 7.2), 10 mm DTT, and 100 mm NaCl. The samples were incubated overnight at 4 °C with gentle rotation, centrifuged for 1 h at 16,900 × *g* at 4 °C, to remove trace amounts of protein aggregates, and quantified (32, 38). Dried lipid film of DiPhPC was dissolved in hexadecane containing 0.1% cholesterol to achieve a total lipid concentration of 12.5 mg/ml, and a planar bilayer of DiPhPC was formed by painting the lipid across a 150-μm aperture of a polysulfone cup. 10 mm HEPES buffer (pH 7.4) containing 5 mm CaCl_2_ (1, 51) and 1 m KCl were added on both the *cis* and *trans* sides of the DiPhPC membrane, and the potential across the membrane was maintained using Ag/AgCl electrodes, 1 m KCl, and 2% (w/v) agarose bridges. Freshly folded protein supplemented with 0.1% cholesterol and 1% Triton X-100 was incubated on ice for 20 min, and 0.5–1.0 μl of this solution was added to the *cis* side of the chamber. Channel insertion was achieved by gently stirring the solution. Multichannel measurement required ∼20–100 channels, whereas 1–3 channels were retained for single-channel experiments. A triangular voltage ramp between 0 and ±60 mV at a 3 mV/s ramp rate was used to study the voltage dependence and gating characteristics of hV2^WT^ and its variants. Channel recording was done with a filtering frequency of 400 Hz for single-channel measurements (1–3 channels) and 15 kHz for multichannel (∼20–100 channels) measurements (38, 52). Different filtering frequencies were used to obtain optimal signal-to-noise and to avoid overestimates of the number of channels (53). *G*/*G*_max_ calculations were done for the opening arm of each multichannel recording by considering the mean conductance between 3.0 and 5.0 mV as *G*_max_.

### Yeast survival assays

*Saccharomyces cerevisiae* POR1 deleted (*por1*Δ) yeast strain, BY4742 (MATα, his3Δ1, leu2Δ0, lys2Δ0, ura3Δ0, por1::kanMX4; kind gift from Vito de Pinto, University of Catania, Italy) was used for the study. hV2^WT^, human VDAC isoform 1 (hV1^WT^), and the engineered barrel mutants were cloned in 2 μ multicopy pYX212 shuttle vector carrying the URA3 gene under the control of the constitutive promoter TPI1. The *por1*Δ strain was transformed with either the pYX212 EV or pYX212 carrying hV2^WT^, hV1^WT^, or the mutants, using lithium acetate. Transformed cells were confirmed by colony PCR using pYX212-specific primers and selectively grown on URA-dropout medium. For serial dilution assays, transformed cells were first grown in the URA-dropout medium (0.19% URA dropout powder (w/v), 0.67% yeast nitrogen base (w/v), and 2% glucose (w/v)). BY4742-*por1*Δ control (without the pYX212 vector) was grown in YPD medium (1% yeast extract, 2% peptone, and 2% glucose). The cells were harvested in the early to mid-log phase of the yeast growth cycle (*OD*_600_ ≈ 1.0) and washed twice with freshly autoclaved milliQ water. Cell density was normalized to a final concentration of ∼3 × 10^7^ cells/ml and spotted by drop dilution (10-fold serial dilution) on 2% agar plates containing YPD, YPY (1% yeast extract, 2% peptone, and 2% glycerol), or YPDA (YPD with 40 mm acetic acid). Plates were incubated at 28 °C for 2 days (YPD) or 4 days (YPY, YPDA). The cells were imaged and densitometry analysis was done using Multi Gauge v2.3 (Fuji Photo Film Co., Ltd.) to obtain a quantitative comparison of the spot densities.

### All-atom molecular dynamics simulations

hV2^WT^ structure predicted using I-TASSER (54) was used for the simulations (32). The protein was aligned in the membrane with input from the Orientation of Proteins in Membranes database (55). The hV2^WT^–lipid assembly was generated using the Bilayer Builder tool of CHARMM-GUI (56, 57).

Each simulation contained one protein molecule inserted into a DMPC bilayer. A 2-nm water layer on either side of the bilayer was used to hydrate the protein–lipid complex, and 100 mm NaCl was added and used additionally to neutralize the charges. Each system had a final box dimension of approximately ∼8.5 nm^3^, containing 1 protein molecule, a 1.5 lipid layer, corresponding to ∼160 lipid molecules (∼80 per leaflet), 19 Na^+^ ions, 20 Cl^–^ ions, and ∼9000–11,000 water molecules (32).

V2^18^ was generated *in silico* by deleting residues 283–294, corresponding to strand β19. Similarly, V2^17^ and V2^16^ were generated from hV2^WT^ by deleting residues 263–294 (strands β18–β19) and 252–294 (β17–β19), respectively. The first and last strands for these constructs were allowed to establish hydrogen bonding by simulating the protein–lipid assembly for up to 100 ns. Barrel closure was effected within ∼15–40 ns for the different barrels (Movies SM1–SM3). Attempts were also made to assemble V2^20^ and V2^21^ by duplicating residues 263–275 (corresponding to β18) and 263–294 (corresponding to β18–β19), respectively. However, in the course of the simulation, these additional strands remained membrane-embedded but did not assemble as a part of the barrel. Therefore, further simulations could not be carried out with V2^20^ and V2^21^.

All-atom MDS was carried out using GROMACS v5.0.4 in duplicate using independent starting structures, using parameters reported earlier (32). The temperatures used for the final equilibration and production steps were maintained at 298 K (1 K above the phase transition temperature of DMPC). Every simulation included an energy-minimization step and six equilibration steps wherein restraints were reduced in each step. The final trajectory was generated from a production run of ∼200 ns with zero restraints. All simulations were duplicated using independent assembly files. Analysis of trajectories was carried out using GROMACS v5.0.4, VMD v1.9.2, or PyMOL. The root-mean-square deviation, RMSF, *R*_g_, and SASA were calculated from each independent simulation, as reported earlier (32).

## Data availability

All data are contained within the article.

## Supplementary Material

Supporting Information
