# Supplementary material for: Evolutionary selection of a 19-stranded mitochondrial β-barrel scaffold bears structural and functional significance
Source: J Biol Chem. 2020 Aug 19;295(43):14653–65. doi: 10.1074/jbc.RA120.014366 (PMC7586230; doi:10.1074/jbc.RA120.014366)
Supplement: Supporting Information [file supp_295_43_14653__index.html]

Evolutionary selection of a 19-stranded mitochondrial β-barrel scaffold bears structural and functional significance — Metastable mitochondrial β19 barrel is functionally superior — Evolutionary selection of a 19-stranded mitochondrial β-barrel scaffold bears structural and functional significance — Metastable mitochondrial β19 barrel is functionally superior — Supporting Information 

# Evolutionary selection of a 19-stranded mitochondrial β-barrel scaffold bears structural and functional significance

## Supporting Information

- Supporting Information - SI Figures
- Supporting Information (to be published online) - Movie file 1
- Supporting Information (to be published online) - Movie file 2
- Supporting Information (to be published online) - Movie file 3
