# Supplementary material for: Evolutionary selection of a 19-stranded mitochondrial β-barrel scaffold bears structural and functional significance
Source: J Biol Chem. 2020 Aug 19;295(43):14653–65. doi: 10.1074/jbc.RA120.014366 (PMC7586230; doi:10.1074/jbc.RA120.014366)
Supplement: Supporting Information [file supp_RA120.014366_160852_2_supp_582010_qf3cm8.pdf]

Evolutionary selection of a 19-stranded mitochondrial β-barrel scaffold bears structural and functional significance

Shashank Ranjan Srivastava[1] and Radhakrishnan Mahalakshmi[1,*]

[1]Molecular Biophysics Laboratory, Department of Biological Sciences, Indian Institute of Science Education and Research, Bhopal – 462066. India.

*Corresponding author. E-mail: maha@iiserb.ac.in.

Supporting Information

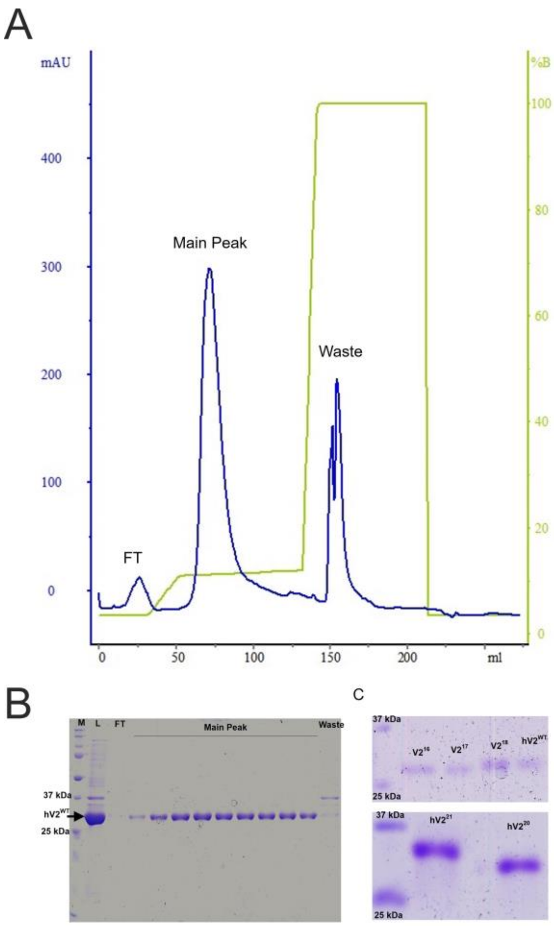

Figure S1. Purification of hV2^{WT} and the engineered barrel variants. (A) Representative purification profile of hV2^{WT} obtained on a HiPrep Q-FF ion exchange column (GE Healthcare) using Buffer A (20 mM Tris-HCl pH 9.5 containing 8.0 M urea) and Buffer B (Buffer A + 1 M NaCl) on a fast protein liquid chromatography system. The absorbance at 280 nm (blue graph, left axis) was used to monitor protein elution at different NaCl concentrations (gradient used is represented as a green curve, left axis). hV2^{WT} and all its variants eluted at 10-11% NaCl (marked as main peak), except V2^{16}, which eluted at 10-12% NaCl. FT: unbound flow through; Waste: tightly bound *E. coli* proteins. (B, left) Image of 12% SDS-PAGE showing protein constituents from the various fractions of the chromatographic profile (M: molecular weight marker; L: load). (B, right) Representative 12% SDS-PAGE images of purified V2^{16}, V2^{17}, V2^{18} and hV2^{WT} (top), V2^{20} and V2^{21} (bottom) used for various experiments.

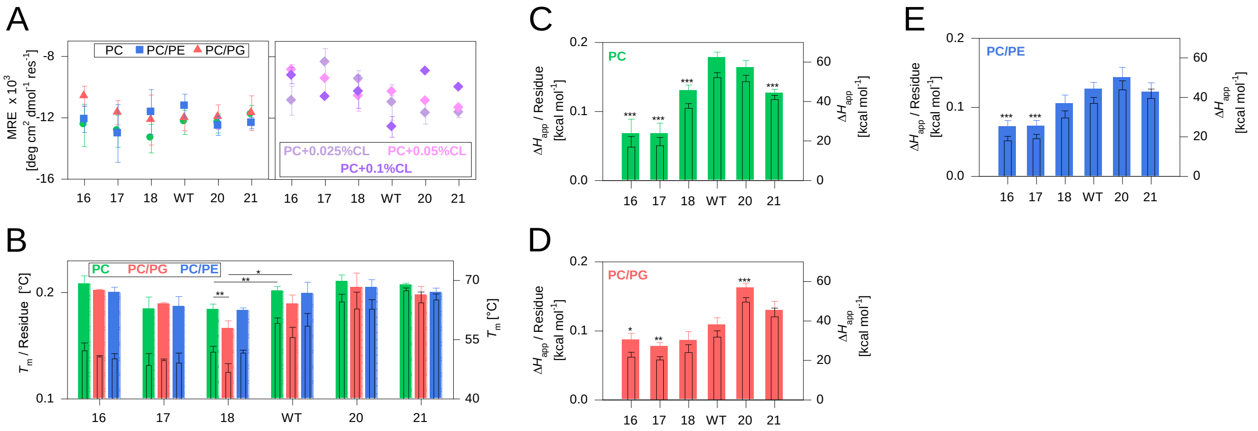

Figure S2. Secondary structure content and T_m of hV2^{WT} and the engineered barrel variants in different lipid bicelles. (A) Far-UV circular dichroism (CD) wavelength scans were obtained from 203 nm – 260 nm at 1 nm increments, and raw ellipticity values were converted to mean residue ellipticity (MRE). The average MRE between 215-220 nm was compared for hV2^{WT} and the engineered variants in different lipid conditions. (A, left) MRE obtained for DMPC (PC), DMPC/DMPE (PC/PE) and DMPC/DMPG (PC/PG). (A, right) MRE obtained for DMPC doped with various percentages of cardiolipin (CL) (0.025%, 0.05%, and 0.1%). Error bars in both graphs represent the standard deviation derived from 3-5 independent experiments. Color codes used for the various lipid conditions are shown within the respective plots. 16: V2^{16}, 17: V2^{17}, 18: V2^{18}, WT: hV2^{WT}, 20: V2^{20}, 21: V2^{21}. (B) Histograms comparing thermal stabilities measured as the mid-point of thermal denaturation (T_m) for hV2^{WT} and the engineered barrel variants in various lipid conditions (PC, PC/PG, PC/PE). The per-residue T_m (left axis) is shown as filled histograms, and the absolute T_m (right axis) is shown as hollow bars. T_m was derived by fitting the thermal unfolding profile to a two-state equation (see methods for details). Error bars represent s. d. from 3-5 independent experiments and independent fits. (C-E) (continued from Figure 2B of the main text) Change in unfolding enthalpy (ΔH_{app}) compared for hV2^{WT} and its variants in PC (C), PC/PG (D), and PC/PE (E). Statistical analysis in B-E was carried out using a two-tailed t-test (*, P≤ 0.05; **, P≤ 0.01; ***, P≤ 0.002), and are presented with reference to hV2^{WT} in C-E.

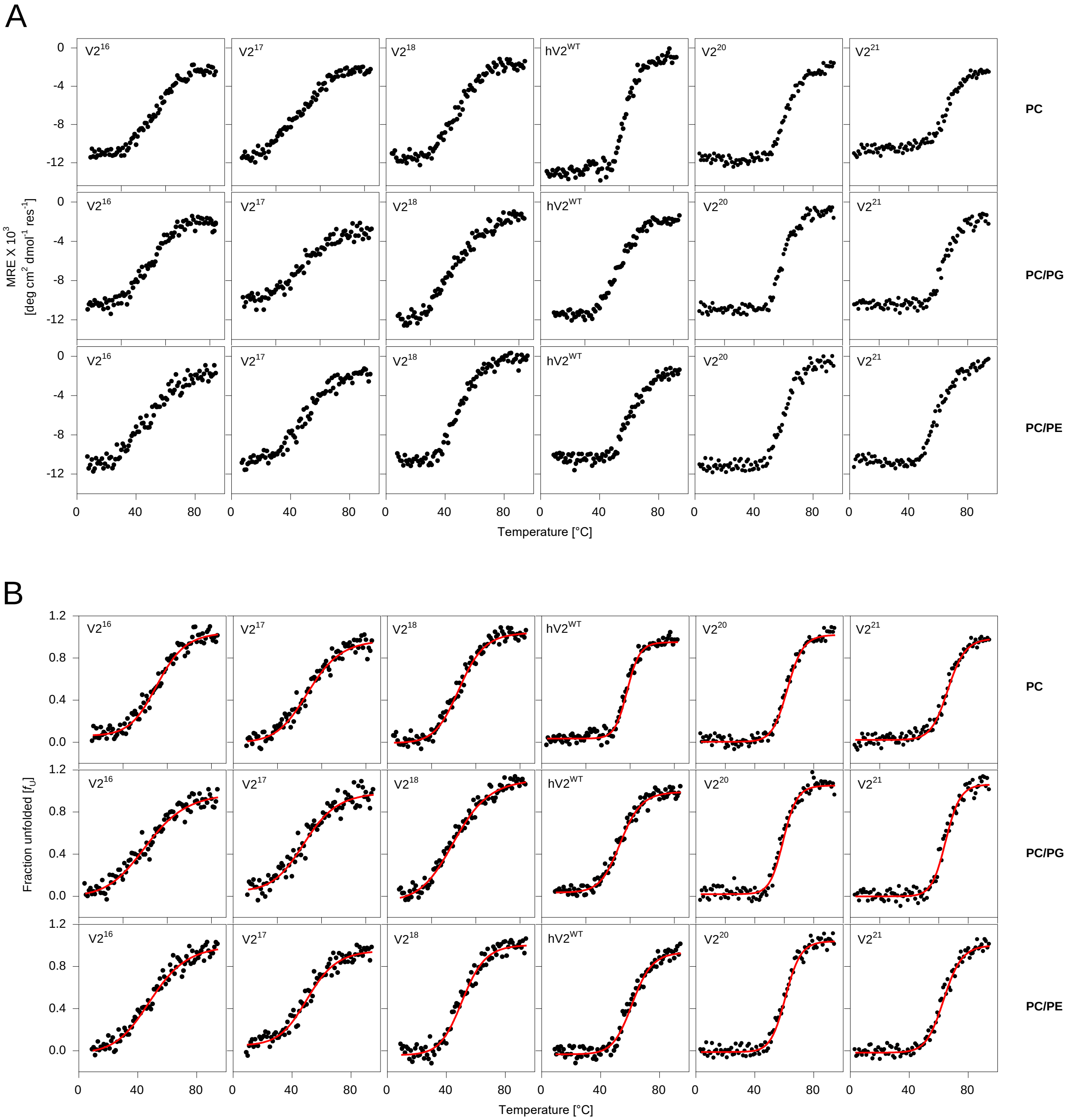
A
V2^16
V2^17
V2^18
hV2^WT
V2^20
V2^21
PC
PC/PG
PC/PE
MRE X 10^3
[deg cm^2 dmol^-1 res^-1]
0
-4
-8
-12
40
80
Temperature [°C]
B
Fraction unfolded [f_U]
1.2
0.8
0.4
0.0

C

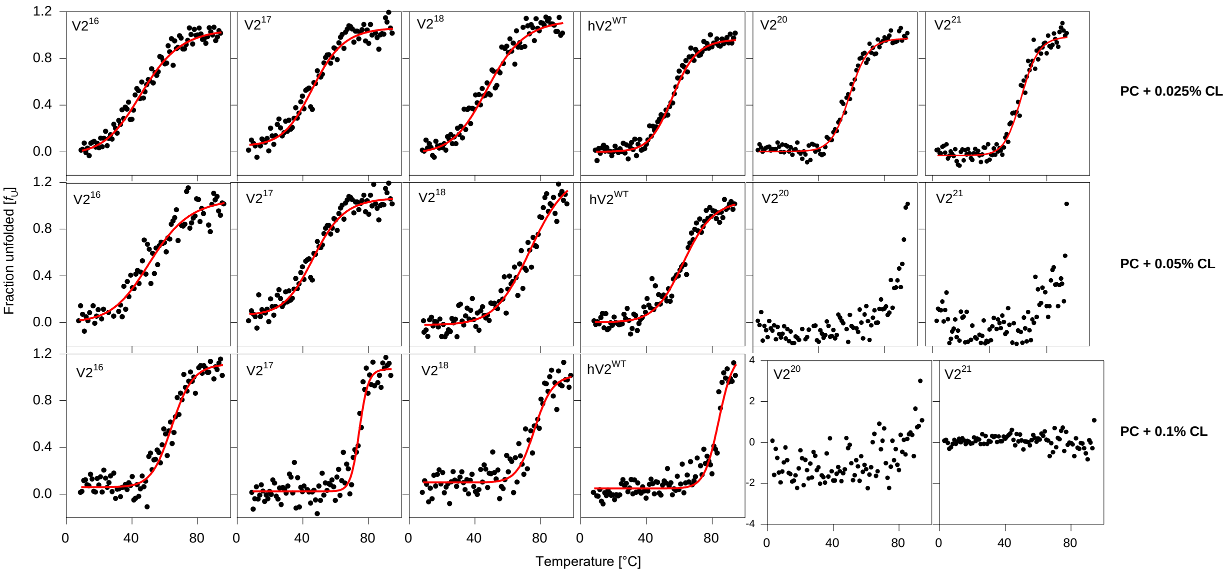

Figure S3. Representative thermal denaturation profiles for hV2^{WT} and the engineered barrel variants in different lipid bicelles. Representative unfolding profiles for the barrel variants obtained by monitoring thermal denaturation from 4 °C – 95 °C, using far-UV CD at 215 nm. Data were normalized between 0 and 1 to obtain the fraction unfolded at each temperature, and fitted to a two-state equation (fits are shown as solid red lines). (A, B) Data obtained from different lipid conditions: DMPC (PC), DMPC/DMPG (PC/PG) and DMPC/DMPE (PC/PE) are shown as raw data (MRE) in (A) and as normalized values in (B). (C) Data obtained in DMPC bicelles doped with various % of CL (0.025%, 0.05% and 0.1%). Data could not be fitted for V2^{20} and V2^{21} in 0.05% and 0.1% CL, as protein unfolding was incomplete even at 95 °C.

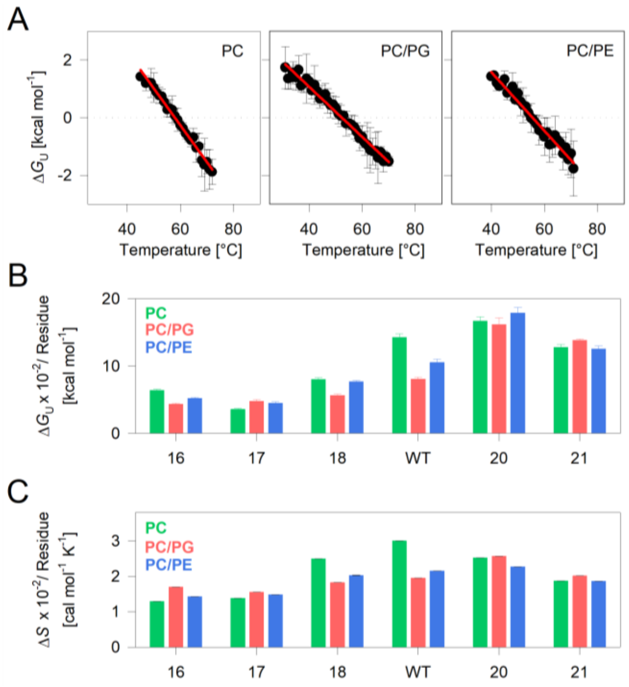

Figure S4. Comparison of thermal parameters derived for hV2^{WT} and the engineered variants. (A) Change in unfolding free energy (ΔG_U) with temperature ($\Delta G_U(T)$) was calculated from the thermal unfolding profiles shown in Figure S3B. Here, $\Delta G_U(T) = -RT \ln(f_U/(1-f_U))$, where f_U is the unfolded fraction at temperature T (see Figure S3B). The data (for the unfolding transition) were fitted to a linear function (fits shown in red) and extrapolated to obtain the apparent ΔG_U at 25 °C. Shown here is the data for hV2^{WT} as the representative barrel in all three lipidic conditions. Comparison of the per-residue apparent ΔG_U at 25 °C (B) and the per-residue ΔS at T_m (C) in all three lipidic conditions across the various hV2 variants. ΔS was calculated as apparent $\Delta G_U = \Delta H_{app} - T_m\Delta S$. At T_m, the apparent $\Delta G_U = 0$. The results show that the apparent ΔG_U follows a similar trend as the ΔH_{app} obtained directly from the thermal unfolding profiles. Overall, we conclude that hV2^{WT} stability is high in DMPC, and this 19-stranded barrel responds by changing its stability upon addition of DMPE or DMPG.

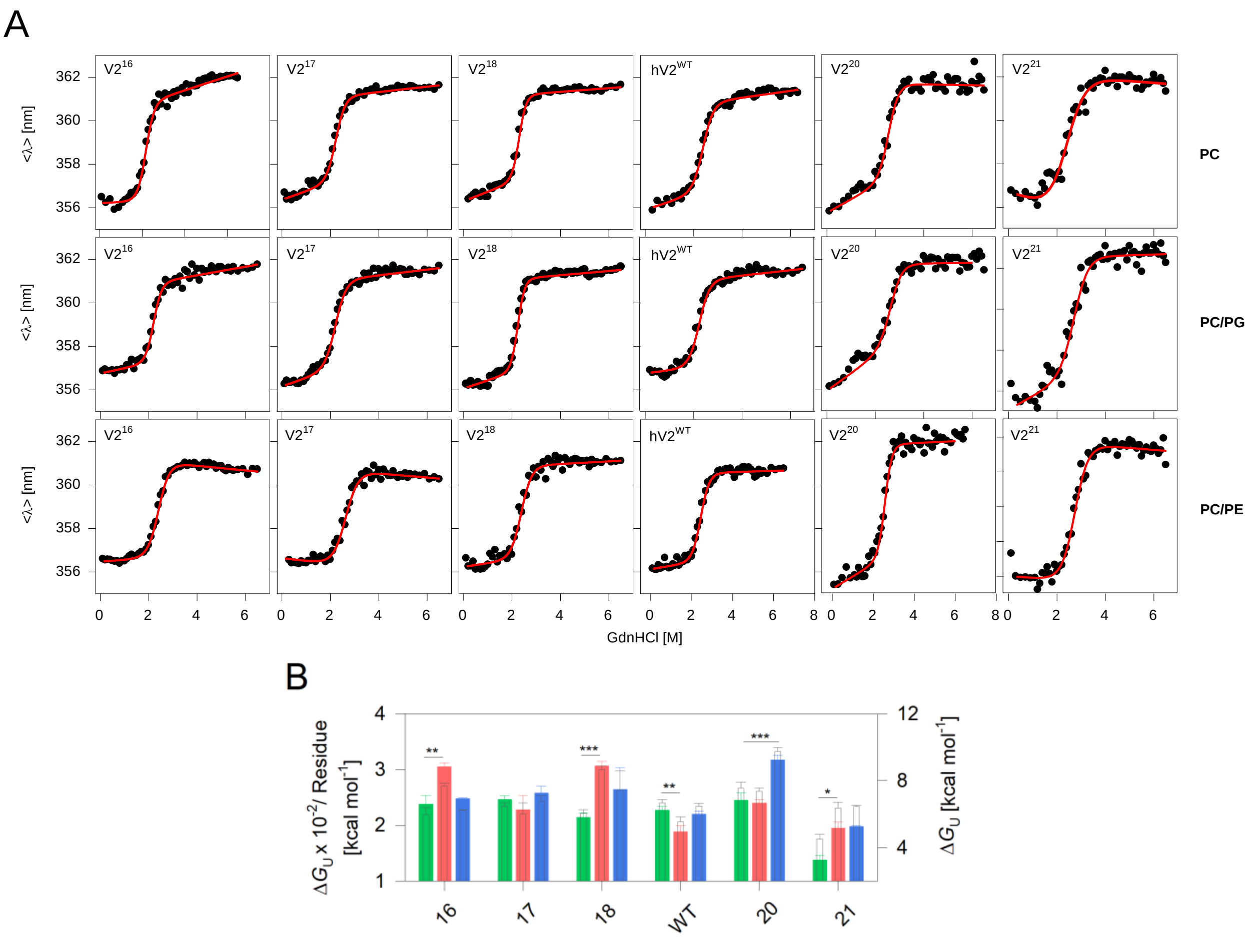

Figure S5. Equilibrium unfolding profiles and ΔG_{eq} for hV2^{WT} and the engineered barrel variants in different lipid bicelles. (A) Weighted mean values (<λ>) for fluorescence emission intensities of the four intrinsic tryptophans, measured from 320 nm – 400 nm, are plotted at different concentrations of the denaturant (guanidine hydrochloride; GdnHCl). Shown here is a representative unfolding profile obtained for each protein in DMPC (PC), DMPC/DMPG (PC/PG), and DMPC/DMPE (PC/PE) lipid bicelles. Fits of the data to a two-state equation (see methods for details) to derive the ΔG_{eq} (plotted in (B)) are shown as solid red lines. (B) Histograms comparing the per-residue ΔG_{eq} (left axis, filled histograms) and total ΔG_{eq} (right axis, hollow bars) across the three lipid conditions (PC, green; PC/PG, red; PC/PE, blue). Error bars represent s. d. from 3-5 independent experiments fitted independently. Statistical analysis was carried out using a two-tailed t-test (*, $P \leq 0.05$; **, $P \leq 0.01$; ***, $P \leq 0.002$).

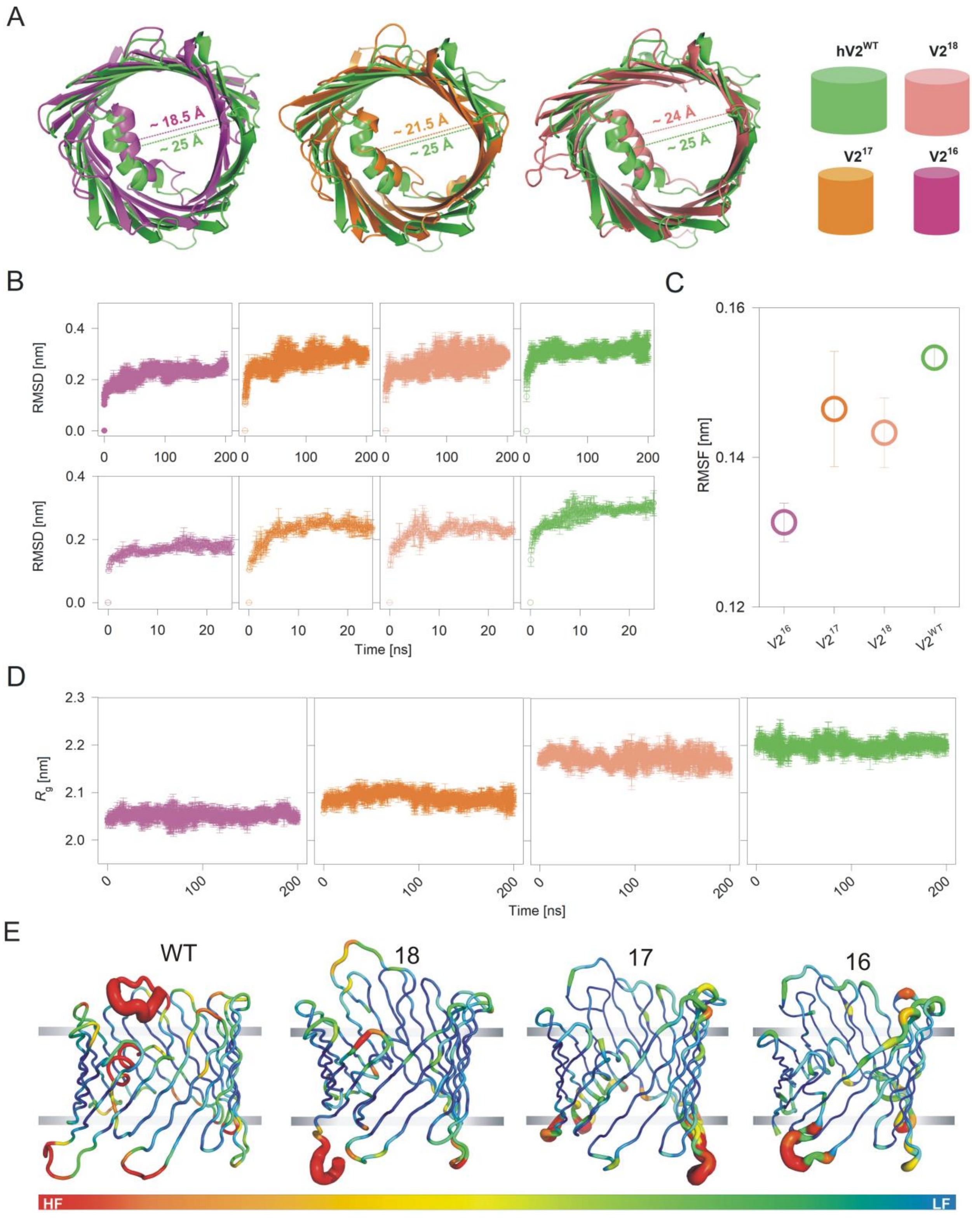

Figure S6. Physico-chemical characteristics of $hV2^{WT}$ and the barrel variants deduced from all-atom MDS. (A) Barrel structure (averaged from 10ns – 200 ns all-atom molecular dynamics simulations) of $V2^{16}$ (left; magenta), $V2^{17}$ (middle; orange) and $V2^{18}$ (right; pink) overlaid on

hV2^{WT} (green), shown as a top view (as seen from the cytosolic side). Strand deletion is accompanied by a reduction in the pore dimensions (indicate within each structure). (B) Root mean square deviation (RMSD) plot for V2^{16}, V2^{17}, V2^{18} and hV2^{WT} for a 200 ns simulation in a DMPC lipid bilayer. The lower panel represents RMSD obtained for the first 25 ns. Note how the barrel attains the end state within 5 ns of the simulation (indicated by no further change in RMSD). Therefore, all calculations were carried out using data from 10 ns – 200 ns. Error bars represent the s. d. obtained from two independent simulations. (C) Per residue root mean square fluctuation (RMSF) derived from the 10-200 ns simulation for residues 33-294. Note how the RMSF is higher for barrel variants V2^{17} and hV2^{WT}, indicating that odd-stranded scaffold variants exhibit higher plasticity compared to the even-stranded barrels V2^{16} and V2^{18}. (D) Radius of gyration (R_g) for V2^{16}, V2^{17}, V2^{18} and hV2^{WT} calculated from the 10-200 ns simulation results in DMPC bilayers. R_g for V2^{16} is comparable to V2^{17}. Similarly, R_g for V2^{18} and hV2^{WT} are analogous. However, a distinct difference in R_g is observed between V2^{17} and V2^{18}. The variation in R_g between odd- and even-stranded barrels is therefore non-linear. (E) Sausage representation illustrating the local fluctuations (dynamics) for the barrel variants in DMPC, obtained throughout the 10 ns – 200 ns simulation run. Color gradient represents high (red) to low (blue) fluctuation.
